# Evaluating the Genetic Effects of Gut Microbiota on the Development of Neuroticism and General Happiness: A Polygenic Score Analysis and Interaction Study Using UK Biobank Data

**DOI:** 10.3390/genes14010156

**Published:** 2023-01-06

**Authors:** Yumeng Jia, Shiqiang Cheng, Li Liu, Bolun Cheng, Chujun Liang, Jing Ye, Xiaomeng Chu, Yao Yao, Yan Wen, Om Prakash Kafle, Feng Zhang

**Affiliations:** Key Laboratory of Trace Elements and Endemic Diseases of National Health and Family Planning Commission, School of Public Health, Health Science Center, Xi’an Jiaotong University, Xi’an 710061, China

**Keywords:** gut microbiota, polygenic risk scores, neuroticism, general happiness

## Abstract

Limited efforts have been invested in exploring the interaction effects between genetic factors and gut microbiota on neuroticism and general happiness. The polygenic risk scores (PRS) of gut microbiota were calculated from individual-level genotype data of the UK Biobank cohort. Linear regression models were then used to assess the associations between individual PRS of gut microbiota and mental traits and interaction analysis was performed by PLINK2.0. KOBAS-i was used to conduct gene ontology (GO) enrichment analysis of the identified genes. We observed suggestive significant associations between neuroticism and PRS for the genus *Bifidobacterium* (rank-normal transformation, RNT) (beta = −1.10, *P* = 4.16 × 10^−3^) and the genus *Desulfovibrio* (RNT) (beta = 0.54, *P* = 7.46 × 10^−3^). PRS for the genus *Bifidobacterium* (hurdle binary, HB) (beta = 1.99, *P* = 5.24 × 10^−3^) and the genus *Clostridium* (RNT) (beta = 1.26, *P* = 9.27 × 10^−3^) were found to be suggestive positively associated with general happiness. Interaction analysis identified several significant genes that interacted with gut microbiota, such as *RORA* (rs575949009, beta = −45.00, *P* = 1.82 × 10^−9^) for neuroticism and *ASTN2* (rs36005728, beta = 19.15, *P* = 3.37 × 10^−8^) for general happiness. Our study results support the genetic effects of gut microbiota on the development of neuroticism and general happiness.

## 1. Introduction

Mental disorders are highly prevalent, placing a heavy burden on global public health [1]. The estimated prevalence of mental disorders was 0.97 billion globally in 2017 [2]. According to a recent report, mental disorders have consistently accounted for more than 14% of age-standardized years lived with disability for nearly three decades [2]. Neuroticism and positive emotion are common personality traits that are often of concern [3,4]. Mental traits are the product of the interplay between genetic and environmental influences. The estimated heritability was 40~60% for neuroticism and 37~64% for positive emotion [5,6]. With regards to environmental influences, adversities during development, such as “emotional neglect and sexual abuse”, were found to be positively associated with these two common mental traits [5,7,8]. However, the pathogenesis of mental traits remains elusive even now.

Gut microbiota co-develops with the host from birth and changes throughout growth depending on different physiological, pathological, dietary patterns, and environmental conditions [9]. Increasing evidence has indicated that the gut microbiota is closely related to host health and plays an important role in the etiology of a variety of complex human disorders, including mental illness [10,11,12]. Cryan et al. suggested that the gut microbiota is a pivotal part of the signaling along the microbiota–gut–brain axis, a bi-directional communication network encompassing the gut microbiota and the nervous system, as well as neuroendocrine and neuroimmune pathways [13,14,15]. However, limited efforts have been invested in exploring the impact of the interaction effects between genetic factors and gut microbiota on mental traits.

Genome-wide association studies (GWAS) have successfully revealed single nucleotide polymorphisms that are associated with mental traits. Recently, using fecal 16S ribosomal RNA gene sequences and host genotype data from the Flemish Gut Flora Project (n  =  2223) and two German cohorts (n  =  1667), Hughes et al. [16] identified several genetic associations involving multiple microbial traits, which were described as continuous (relative abundance, α-diversity), binary (presence/absence), multinomial (enterotypes), and multivariate (β-diversity) traits. However, the results obtained by GWAS are usually derived from individual causal loci and the effect sizes are relatively weak [17]. To overcome this difficulty, polygenic risk scores (PRS) has been used to predict the risk of human disease, which is a sum of trait-associated alleles across many genetic loci, typically weighted by effect sizes estimated from an independent large-scale discovery GWAS [18]. More importantly, since there are no available gut microbiota data for mental traits in UK Biobank, utilizing microbiota-based PRS can be used to explore the relationship between emotional traits and gut microbiota. By using microbiota-based PRS, previous studies have explored the effects of gut microbiota interactions with C-reactive protein or brain aging on psychiatric disorders such as depression and anxiety [19,20]. Until now, precise association loci and interactions effects between gut microbiota on the development of neuroticism and general happiness remain unidentified despite the role of the gut microbiota on the etiology of mental disorders having already been demonstrated. 

In this study, we first calculated the PRS of gut microbiota in the UK Biobank cohort in relation to emotional traits. Briefly, linear regression analyses were first performed to detect the associations between individual PRS values of gut microbiota and the phenotypic data of neuroticism and general happiness in the UK Biobank cohort. Finally, interaction analysis was conducted to explore candidate gene–gut microbiota interactions on the development of mental traits. The results of this study may expand our genetic understanding of the effect of the gut microbiota on these two mental traits.

## 2. Materials and Methods

### 2.1. UK Biobank Dataset

The UK Biobank study is a large prospective cohort study including health data, hospital records, and genetic data from 502,656 participants aged 40–69 in 2006 and 2010 [21]. UK Biobank received electronically signed consent from the study participants, and ethical approval was obtained from the Northwest Multi-Centre Research Ethics Committee (reference 11/NW/0382). We used the imputed genotype dataset made available by the UK Biobank in its July 2017 release. We restricted participants to “white British” individuals based on self-reported ethnicity. Subjects who had a self-reported sex inconsistent with the genetic sex, who were genotyped but not imputed, or who withdraw their consents were removed. After removing the participants without the calculated gut microbiota-related PRS, 306,161 participants for G_*Bifidobacterium*_RNT and neuroticism, 153,483 participants for G_*Desulfovibrio*_RNT and neuroticism, 89,206 participants for G_*Bifidobacterium*_HB and general happiness, and 115,013 participants for G_*Clostridium*_sensu_stricto_RNT and general happiness were included for association analysis. All participants agreed to allow the use of their anonymous data to conduct any health-related studies and to reconnect for further sub-studies. 

Genotyping, quality control, and imputation were performed by the UK Biobank. DNA samples of all participants in the UK Biobank were genotyped using either the Affymetrix UK BiLEVE (807,411 markers) or Affymetrix UK Biobank Axiom (825,927 markers) array [22]. Single-nucleotide polymorphisms (SNPs) were imputed by IMPUTE2 against the reference panel of the Haplotype Reference Consortium, 1000 Genomes, and UK10K projects. Full details regarding these data are available elsewhere [23]. This research has been conducted using the UK Biobank Resource under Application Number 46478. The authors thank all UK Biobank participants and researchers who contributed or collected data.

### 2.2. Phenotypes Definition 

Neuroticism score is derived based on 12 domains of neurotic behavior as reported from UK Biobank data fields 1920, 1930, 1940, 1950, 1960, 1970, 1980, 1990, 2000, 2010, 2020, and 2030 from the touchscreen questionnaire at baseline. Participants were assessed for the 12 domains of neurotic behaviors via the touchscreen questionnaire. Neuroticism score summarizes the number of “Yes” answers across the 12 questions into a single integer score for each participant. The detailed questionnaire is shown in Supplementary File S1. General happiness was collected from the response to the UK Biobank online “Thoughts and Feelings” mental health questionnaire: “In general how happy are you?” by choosing “Do not know (−121)”, “Extremely happy (1)”, “Very happy (2)”, “Moderately happy (3)”, “Moderately unhappy (4)”, “Very unhappy (5)”, “Extremely unhappy (6)”, and “Prefer not to answer (−818)”. The subjects whose answers are “Do not know (−121)” and “Prefer not to answer (−818)” were excluded from this study. The detailed definition of phenotypes is shown in Supplementary File S1. 

### 2.3. GWAS Summaries of Gut Microbiota 

The gut microbiota-associated SNPs were derived from a recently published GWAS [16]. Briefly, using fecal 16S ribosomal RNA gene sequences and host genotype data from the Flemish Gut Flora Project (n  =  2223) and two German cohorts (n  =  1667), Hughes et al. [16] identified several genetic associations involving multiple microbial traits. Sequencing was carried out on the Illumina HiSeq platform at the VIB Nucleomics core laboratory (Leuven, Belgium), with 500 cycles (sequencing kit HiSeq-Rapid SBS v.2). After merging paired sequences and the removal of chimeras, compositional matrices for each taxonomical level were carried out using the Ribosomal Database Project (RDP) training set ‘rdp_train_set_16’. Microbial taxa were described as relative abundance profiles using the rank normal transformed (RNT) model, while those with zero-inflated abundance distributions were described using a hurdle binary (HB) model. For association analysis, any taxa that met the following two criteria were included: (1) comprise ≥5% of the reads for at least one individual; and (2) have ≥15% of individuals with non-zero data were included. In total, 114 taxa across all phylogenetic levels met these criteria. We selected microbial traits associated with SNPs with a significant threshold of *P* < 5 × 10^−6^ to calculate the PRSs. A detailed description of sample characteristics, array design, quality control, and statistical analysis can be found in the previous study [16]. 

### 2.4. PRS Analysis

Using the genotype data of the UK Biobank cohort, PRS analysis was performed by using the PLINK’s “-score” command [24]. Briefly, let *PRSg* denote the PRS value of each microbial trait for the *g*th subjects, where *i* (i = 1, 2, 3, …, l) and *g* (g = 1, 2, 3, …, k) denote the number of genetic markers and sample size, respectively. *βi* is the effect parameter of the risk allele of the *i*th significant SNP related to each microbial trait obtained from the previously published study. *SNPig* is the dosage (0 to 2) of the risk allele of the *i*th SNP for the *g*th subject. The PRS values were standardized to have a mean of 0 and a variance of 1 before further analyses. Using computed PRSs as the instrumental variables of gut microbiota, a linear regression model was finally used to detect the potential associations between gut microbiota and target traits. In this study, the significant association thresholds should be *P* < 2.19 × 10^−4^ [0.05/(114 × 2)] after strict Bonferroni correction. The suggestive significance threshold was set as *P* < 0.05. All statistical analyses were performed using R3.5.3 (https://www.r-project.org/ (accessed on 5 February 2021)). Additionally, the sex, age, and 10 principal components of the population structure were used as covariates in the regression model. 

### 2.5. Statistical Analysis

Based on the result of the regression model, interaction analysis was then conducted to explore the impact of the interaction between genetic factors and gut microbiota traits related to PRS on mental traits in the UK Biobank cohort. The outcome variables, including neuroticism and general happiness, were adjusted by sex, age, and 10 principal components of population structure. The instrumental variables are the PRS of gut microbiota traits. Based on the previous study, the genetic additive (ADD) model of PLINK2.0 was used in this study [25]. SNPs with a call rate <0.95, Hardy–Weinberg equilibrium testing of *P* < 0.001, and minor allele frequencies (MAFs) < 0.01 were excluded for quality control [25]. A significant threshold was set at *P* = 5.0 × 10^−8^. Circular Manhattan plots were generated using the “CMplot” R script (https://github.com/YinLiLin/R-CMplot (accessed on 3 April 2021)).

### 2.6. Gene Set Enrichment Analyses

To explore the functional relevance of identified genes interacting with individual PRS of gut microbiota for neuroticism and general happiness, gene ontology (GO) enrichment analyses of the identified target genes were performed by using KOBAS-i (short for KOBAS intelligent version) (http://kobas.cbi.pku.edu.cn/ (accessed on 29 December 2022) [26]. 

## 3. Result

### 3.1. Basic Characteristics of Study Samples

The general characteristics of the subjects are presented in Table 1. For the association analysis of neuroticism and G_*Bifidobacterium*_RNT, 306,161 participants were selected; 161,977 of them were female, with mean age (SD) was 56.81 (7.93) years. For the association analysis of neuroticism and G_*Desulfovibrio*_RNT, 153,483 participants were selected; 81,310 of them were female, with mean age (SD) was 56.80 (7.93) years. For the association analysis of general happiness and G_*Bifidobacterium*_HB, 89,206 participants were selected; 49,806 of them were female, with mean age (SD) was 56.27 (7.62) years. For the association analysis of general happiness and G_*Clostridium*_sensu_stricto_RNT, 115,013 participants were selected; 64,229 of them were female, with mean age (SD) was 56.28 (7.61) years.

We observed suggestive significant associations between neuroticism and the PRS of the genus *Bifidobacterium* (RNT) (beta = −1.10, *P* = 4.16 × 10^−3^) and genus *Desulfovibrio* (RNT) (beta = 0.54, *P* = 7.46 × 10^−3^). The PRS of the genus *Bifidobacterium* (HB) (beta = 1.99, *P* = 5.24 × 10^−3^) and the genus *Clostridium* (RNT) (beta = 1.26, *P* = 9.27 × 10^−3^) were found to be positively suggestive associated with general happiness. 

We further compared the above association analysis results, and we found that the genus *Bifidobacterium* was shared by both participants with neuroticism and general happiness, genus *Bifidobacterium* (beta _neuroticism_ = −1.10, *P*_neuroticism_ = 4.16 × 10^−3^; beta _general happiness_ = 1.99, *P* _general happiness_ = 5.24 × 10^−3^)

### 3.2. Interaction Analysis of Gut Microbiota with Mental Traits

**Figure 1:**
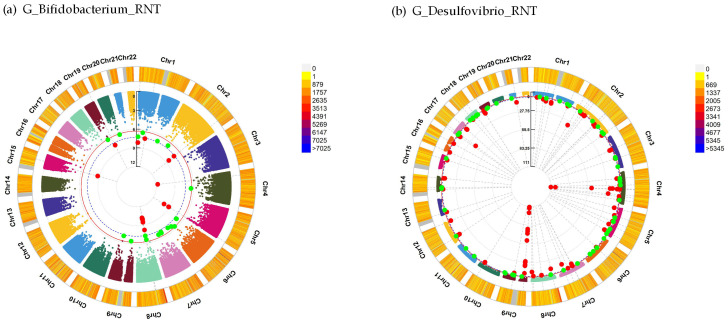
Interaction analysis between SNP and gut microbiota for neuroticism. Chromosomal regions interacting with G_*Bifidobacterium*_RNT (**a**) and G_*Desulfovibrio*_RNT (**b**) for neuroticism. From the center, the first circos depicts the −log_10_ *P* of each variant owing to double exposure, i.e., the effect of both single nucleotide polymorphism allele and gut microbiota. The second circos shows chromosome density. Red plots indicate the *P*-value threshold for genome-wide significance (*P* < 5 × 10^−8^), while the green plots indicate the *P*-value threshold for suggestive significance (*P* < 5 × 10^−7^). The plots were generated using the “CMplot” R script (https://github.com/YinLiLin/R-CMplot (accessed on 3 April 2021)). SNP, single-nucleotide polymorphism; G, genus; RNT, rank-normal transformation.

For neuroticism, the interaction analysis identified 17 significant SNPs interacted with the genus *Bifidobacterium* (RNT) at *P* < 5.0 × 10^–8^, such as *RORA* (rs575949009, beta = −45.00, *P* = 1.82 × 10^−9^) and *NDUFS1* (rs148934517, beta = −15.60, *P* = 3.71 × 10^−8^), and 249 significant SNPs interacted with the genus *Desulfovibrio* (RNT) were identified at *P* < 5.0 × 10^–8^, such as *KCNQ3* (rs58613338, beta = −14.79, *P* = 2.62 × 10^−8^) (Table 2, Figure 1). The detailed significant interaction results (*P* < 5.0 × 10^−8^) for neuroticism are summarized in Table S1.

**Figure 2:**
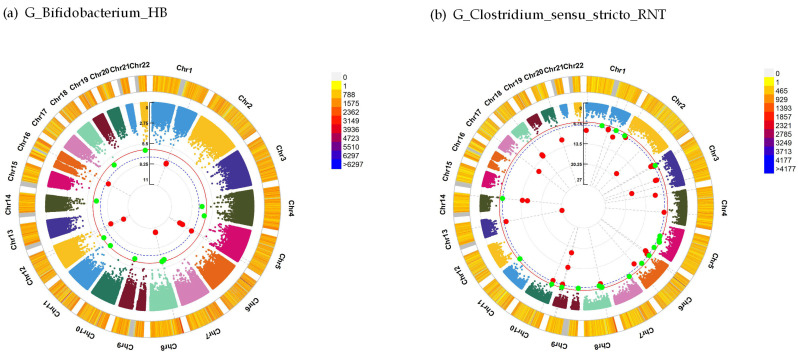
Interaction analysis between SNP and gut microbiota for general happiness. Chromosomal regions interacting with G_*Bifidobacterium*_HB (**a**) and G_*Clostridium*_sensu_stricto_RNT (**b**) for general happiness. From the center, the first circos depicts the –log_10_ *P* of each variant owing to double exposure, i.e., the effect of both single nucleotide polymorphism allele and gut microbiota. The second circos shows chromosome density. Red plots indicate the *P*-value threshold for genome-wide significance (*P* < 5 × 10^−8^), while the green plots indicate the *P*-value threshold for suggestive significance (*P* < 5 × 10^−7^). The plots were generated using the “CMplot” R script (https://github.com/YinLiLin/R-CMplot (accessed on 3 April 2021)). SNP, single-nucleotide polymorphism; G, genus; HB, hurdle binary; RNT, rank-normal transformation.

For general happiness, the interaction analysis found 17 significant SNPs interacted with the genus *Bifidobacterium* (HB) at *P* < 5.0 × 10^–8^, such as *DCTN4* (rs252157, beta = 41.95, *P* = 9.23 × 10^−10^) and *MYOZ3* (rs194134, beta = 43.12, *P* = 4.74 × 10^−10^), and 130 significant SNPs interacted with the genus *Clostridium* (RNT) were identified, such as *ASTN2* (rs36005728, beta = 19.15, *P* = 3.37 × 10^−8^) (Table 3, Figure 2). The detailed significant interaction results (*P* < 5.0 × 10^−8^) for general happiness are summarized in Table S2.

### 3.3. GO Enrichment Analysis Results

GO enrichment analysis identified five GO terms enriched in the significant genes interacting with the genus *Bifidobacterium* (RNT), such as GO:0005515–protein binding (*P* = 8.12 × 10^−3^), and 15 GO terms enriched in the significant genes interacting with the genus *Desulfovibrio* (RNT) for neuroticism, such as GO:0005925–focal adhesion (*P* = 1.56 × 10^−2^), GO:0045202–synapse (*P* = 1.57 × 10^−2^). Fourteen GO terms enriched in the significant genes interacting with the genus *Clostridium* (RNT) for general happiness were identified, such as GO:0045666–positive regulation of neuron differentiation (*P* = 2.46 × 10^−4^). The detailed enrichment analysis results are summarized in Tables S3 and S4.

## 4. Discussion

In this study, we conducted PRS and interaction analyses to explore the relationship between gut microbiota and two common mental traits, neuroticism and general happiness. We found significant associations between gut microbiota and the risk of neuroticism and general happiness. In addition, we identified multiple genes which interacted with the gut microbiota influencing neuroticism and general happiness.

It has been demonstrated by previous studies that the gut microbiota is associated with certain behaviors and psychiatric disorders, which are consistent with our findings. For example, according to an observational and cross-sectional study in 672 adults, high neuroticism was correlated with a high abundance of *Gammaproteobacteria* and *Proteobacteria*, respectively, when age, sex, BMI, and nutrient intake were controlled as covariates. The results of the beta diversity analysis indicate a lower diversity and closer similarity within paired samples in the neuroticism group [27]. Michels et al. suggested that adjusted and unadjusted taxonomic differences in the gut microbiota were the most pronounced for happiness, which was associated with 24 operational taxonomic units (=11.8% of bacterial counts) in 93 Belgian adolescents [28]. Another research from healthy Korean adults revealed that positive emotion and gut microbiome diversity (Shannon Index) were associated with substantial relationships in the *Prevotella*-dominating group [29]. 

We observed a suggestive significant negative correlation between the PRS of the genus *Bifidobacterium* and neuroticism and a positive correlation between the genus *Desulfovibrio* and neuroticism. Although evidence on the genus *Bifidobacterium* and neuroticism is limited, anxiety and depression, typical characteristics among populations with high neuroticism [30], have been shown to be strongly associated with *Bifidobacterium*. Animal experiments have observed that long-term exposure to a 150 mT static magnetic field improved the abundance of *Bifidobacterium* and *Clostridium* and further improved anxiety in C57BL/6J mice [31]. A recent clinical study also confirmed the potential of *Bifidobacterium* in the treatment of major depression [32]. They found that oral administration of freeze-dried *Bifidobacterium* breve CCFM1025 significantly reduced the serum serotonin turnover in patients compared to the placebo [32]. The genus *Desulfovibrio* is anaerobic and Gram-negative, also known as a sulfate-reducing bacterium. Recently, our group identified that *Desulfovibrio* was significantly related to attention-deficit/hyperactivity disorder (ADHD), autism spectrum disorder (ASD), bipolar disorder (BD), schizophrenia (SCZ), and major depressive disorder (MDD) [33]. As a potentially harmful bacterium, *Desulfovibrio* was found to be enriched in diseased individuals in inflammatory disease models [34,35]. The abundance of *Desulfovibrio* was negatively correlated with short-chain fatty acids (SCFAs), the production of microbial metabolites [34]. SCFAs, such as acetate, butyrate, and propionate, are important immunomodulatory and anti-inflammatory molecules in the gut and have shown promising therapeutic effects on symptoms of depression and anxiety in animal studies [36]. Despite limited research on *Desulfovibrio* and neuroticism, further multi-omics studies integrating metagenomics, transcriptomics and metabolomics may provide the basis for revealing the mechanisms underlying this interaction.

We also observed suggestive significant positive correlations between the PRS of the genus *Bifidobacterium* and the genus *Clostridium* with general happiness. The genus *Clostridium* is Gram-positive, and most of them are non-pathogenic bacteria. A recent comparative study identified a significantly lower relative abundance of *Clostridium* in patients with schizophrenia than in healthy controls [37]. Emerging evidence supported the beneficial role of *Clostridium* in the anti-inflammatory activity, immune protection, and remodeling of the gut microbiome [38,39]. For example, oral administration of Ataining (containing *Clostridium* butyricum, CGMCC0313.1) to gastrectomized patients resulted in reduced early postoperative inflammation, enhanced immune ability, restored intestinal microbiota eubiosis, and increased intestinal SCFAs [38]. Similarly, *Clostridium* butyricum SLZX19-05 treatment of weaned piglets inhibited inflammation levels, remodeled the ileal microbiome and increased propionate production [39]. Since inflammation and immune system activation play an important role in brain function and the development of mental health [40], the above evidence also supports to some extent the relationship between *Clostridium* and general happiness. Collectively, genus *Bifidobacterium* and *Clostridium* are promising candidate psychological gut microbiota that may improve emotional health. These mechanisms may be related to the production of neuroactive substances, as well as anti-inflammatory effects and immune system activation. These findings support the future clinical use of psychobiotics to improve emotional health.

Notably, we found that the PRS of the genus *Bifidobacterium* was suggestive negatively correlated with neuroticism but positively correlated with general happiness. Mechanistically, as a psychobiotic, *Bifidobacterium* has been reported to secrete γ-aminobutyric acid (GABA, the “happy” chemical) [41]. Moreover, supplementation of *Bifidobacterium* could increase tryptophan, a precursor of serotonin (another “happy” chemical) [42]. Neuroticism, a health-related personality factor that includes negative emotions such as anxiety, moodiness, and depression [43], is opposed to the positive trait of general happiness. Taken together, the positive correlation between the genus *Bifidobacterium* and general happiness and the negative correlation between Bifidobacterium and neuroticism can be speculated.

The interaction effects between genetic factors and gut microbiota traits for mental traits remain largely unknown now. The key element of this study is that we conducted an interaction analysis of mental traits and identified multiple loci and candidate genes for the modulation of genetic response to the gut microbiota, which may provide novel insights to help disentangle its underlying etiological mechanisms. 

Interaction analysis identified several candidate genes interacting with gut microbiota for neuroticism, such as *RORA*, *KCNQ3,* and *NDUFS1*. *RORA* is a member of the nuclear hormone receptor superfamily. Genetic association studies have identified that *RORA* is a suggestive gene linked more specifically to the depression facet of neuroticism [44]. In the mouse nervous system, *RORA* is localized in the cerebellum, thalamus, cerebral cortex, suprachiasmatic nucleus, and other structures [45]. As recently demonstrated by multiple studies of animal models, GWA, and linkage studies converging on variants in the *RORA* gene, it may be linked to bipolar disorder and trait depression [46,47]. RORA proteins play an important role in the maintenance of circadian rhythms [48]. Clinically, the disruption of circadian rhythms may contribute to anxiety disorders [49]. In addition, RORA proteins are involved in protecting neurons and glial cells from oxidative stress-induced apoptosis [50], which is a potential mechanism implicated in the pathophysiology of depression and anxiety disorders. Direct evidence for *RORA* and neuroticism, although limited, suggests these physiological roles of RORA protein may support the biological plausibility of its association with neuroticism. Sands et al. revealed that specific gain-of-function variants in *KCNQ3* cause neurodevelopmental disability, autism, and ASD [51]. In addition, Kaminsky et al. suggested that epigenetic alterations in the *KCNQ3* gene may be important in the aetiopathogenesis of bipolar disorder [52]. As a voltage-gated potassium channel, KCNQ is an important regulator of cell membrane excitability [53]. Preclinical studies suggest that the KCNQ channel is a potential target for the treatment of depression and anhedonia [54]. Specifically, KCNQ2 and KCNQ3 form homologous or heterodimers (such as KCNQ2/3 channels), which constitute M-shaped channels regulating nerve excitability [55]. Up-regulation of the KCNQ3 channel restored the hyperactivity of dopamine neurons and reversed depressive behavior in susceptible mice [56]. A genetic variant in the *NDUFS1* gene was reported to be associated with schizophrenia and negative symptoms in Han Chinese subjects [57]. According to a previous study, neuroticism may share common genetic variants with most mental disorders, such as BD and MDD [58]. *NDUFS1* knockdown in neurons reduced complex I affiliation into supercomplexes, resulting in decreased oxygen consumption and increased mitochondrial reactive oxygen species [59]. Further functional studies are warranted to validate the role of those genes in neuroticism.

*ASTN2* encodes a large vertebrate-specific transmembrane protein that is expressed primarily in developing and adult brains. We observed that it interacted with the genus *Clostridium* to influence general happiness. In recent studies on *ASTN2* and general happiness, although very limited, copy number variants of *ASTN2*, both deletions and duplications, have been identified in patients with neurodevelopmental disorders, including ASD, SCZ, ADHD, BD, intellectual disability, and global developmental delay [60,61,62,63]. The hippocampus is thought to be involved in emotional and cognitive regulation [64]. A dataset test of 724 twins and siblings found a non-linear correlation between subjective well-being (SWB) and hippocampal volume, characterized by lower SWB in subjects with relatively smaller hippocampal volume compared to those with medium and high hippocampal volume [65]. A previous GWAS study identified nine SNPs at 9p33 in *ASTN2* that were significantly associated with hippocampal volume [66]. Another GWAS study also identified one novel independent locus that was significantly associated with hippocampal volume lying within the *ASTN2* gene [67]. Emotional or stressful events, on the other hand, are associated with a large release of corticosterone, which rapidly enhances synaptic plasticity [68]. *ASTN2* can play a role in emotional events by regulating synaptic strength through endocytic transport and the degradation of surface proteins [69]. Conclusively, the above studies suggest that *ASTN2* may influence general happiness by altering the hippocampal volume or regulating synaptic strength, which supports our findings.

GO enrichment analysis identified multiple GO terms associated with neuroticism and general happiness. One notable finding is focal adhesion (GO:0005925). It plays an important role in cell migration during brain development. Previous studies suggest that focal adhesion and extracellular matrix receptor interaction pathways are significantly associated with certain facets of neuroticism (i.e., angry hostility and depression) [70]. It has also been reported that focal adhesion is involved in abnormal neurodevelopment in SCZ patients compared with healthy control subjects [71]. Synapse (GO:0045202) is another notable GO term that we found. A recent finding has raised the importance of glypicans in the development and functions of synapses, and the dysfunctions of glypicans may lead to the malfunction of synapses and abnormal neurodevelopmental disorders, such as neuroticism and SCZ [72].

It is important to emphasize that our study has certain limitations. First, like GWAS, some significant SNPs found by the interaction analysis are located in the non-coding region, which still poses a challenge for us to better illustrate our results. Second, all subjects in this study are of European ancestry. Therefore, one should be careful when applying our study results to other ethnic groups. Next, because the use of antibiotics may disrupt the composition of the microbiota [73], future studies to explore the impact of antibiotic use on mental traits by affecting gut microbiota are warranted. Further experiments are needed to confirm our findings and reveal the potential molecular mechanisms underlying the associations observed in this study. Finally, the gut microbiota-related SNP sets were derived from previous GWAS. The accuracy of our findings may be influenced by the power of the previous study. Further replication studies with other genetic background individuals and experimental studies are required to verify the results of this study. 

## 5. Conclusions

In conclusion, we observed correlations between gut microbial traits and neuroticism and general happiness in the UK Biobank cohort. Interaction analysis identified multiple candidate genes which may serve as the underlying genetic mechanisms of the observed association. Our study findings could provide novel insights into the impacts of gut microbial traits on the two common mental traits.

## Figures and Tables

**Table 1:** The associations between gut microbiota traits and mental traits.

Mental Traits	Gut Microbiota Traits	Sample Size	Sex (Female)	Age ± SD	Beta	SE	R^2^	*P*
Neuroticism	G_*Bifidobacterium*_RNT	306,161	161,977	56.81 ± 7.93	−1.10	0.38	4.16 × 10^−3^	4.16 × 10^−3^
	G_*Desulfovibrio*_RNT	153,483	81,310	56.80 ± 7.93	0.54	0.20	7.46 × 10^−3^	7.46 × 10^−3^
General happiness	G_*Bifidobacterium*_HB	89,206	49,806	56.27 ± 7.62	1.99	0.71	5.23 × 10^−3^	5.24 × 10^−3^
	G_*Clostridium*_sensu_stricto_RNT	115,013	64,229	56.28 ± 7.61	1.26	0.49	9.27 × 10^−3^	9.27 × 10^−3^

Note: G, genus; RNT, rank-normal transformation; HB, hurdle binary; SD, standard deviation; SE, standard error.

**Table 2:** Summary of interaction analysis between SNP and gut microbiota for neuroticism.

	Chromosome	Gene	Beta	*P*
G_*Bifidobacterium*_RNT	5	*GRK6*	−19.43	1.10 × 10^−10^
	2	*NDUFS1*	−15.60	3.71 × 10^−8^
	5	*NSD1*	−6.28	7.91 × 10^−10^
	15	*RORA*	−45.00	1.82 × 10^−9^
G_*Desulfovibrio*_RNT	15	*ARNT2*	−6.40	7.16 × 10^−10^
	14	*CCDC85C*	−12.94	7.27 × 10^−10^
	4	*CCNG2*	−9.75	2.73 × 10^−8^
	19	*CERS4*	−7.55	1.21 × 10^−10^
	13	*FLT3*	−4.94	8.55 × 10^−10^
	8	*KCNQ3*	−14.79	2.62 × 10^−8^
	4	*LAMTOR3*	−13.25	2.09 × 10^−50^
	4	*MANBA*	−9.25	1.18 × 10^−11^
	8	*MSR1*	5.15	1.47 × 10^−15^
	4	*NR3C2*	−13.59	1.49 × 10^−8^
	12	*PPFIBP1*	−3.43	3.27 × 10^−13^
	1	*PRSS38*	−6.85	2.76 × 10^−9^
	2	*RAMP1*	−7.73	6.59 × 10^−11^
	12	*RIMBP2*	−5.15	4.32 × 10^−11^
	4	*SLC9B1*	−10.70	3.01 × 10^−12^
	7	*TPST1*	−15.90	5.34 × 10^−10^
	4	*UBE2D3*	−9.78	3.03 × 10^−12^
	19	*ZNF317*	−12.96	1.01 × 10^−12^

Note: SNP, single-nucleotide polymorphism; G, genus; RNT, rank-normal transformation.

**Table 3:** Summary of interaction analysis between SNP and gut microbiota for general happiness.

	Chromosome	Gene	Beta	*P*
G_*Bifidobacterium*_HB	5	*DCTN4*	41.95	9.23 × 10^−10^
	12	*MAP1LC3B2*	−15.80	6.11 × 10^−9^
	5	*MYOZ3*	43.12	4.74 × 10^−10^
	16	*PRSS54*	−11.58	2.38 × 10^−8^
	1	*RGS21*	14.30	1.67 × 10^−8^
G_*Clostridium*_sensu_stricto_RNT	9	*ASTN2*	19.15	3.37 × 10^−8^
	1	*ATF6*	8.28	2.54 × 10^−8^
	6	*BMP6*	7.55	1.98 × 10^−9^
	1	*CAMTA1*	9.60	8.80 × 10^−10^
	3	*CCDC14*	13.67	1.72 × 10^−9^
	3	*ECT2*	14.69	2.17 × 10^−18^
	2	*FMNL2*	7.78	1.66 × 10^−19^
	1	*LAMB3*	10.86	4.61 × 10^−10^
	3	*MYLK*	13.61	3.95 × 10^−9^
	17	*RHBDL3*	8.91	3.35 × 10^−16^
	9	*SVEP1*	12.88	5.36 × 10^−19^

Note: SNP, single-nucleotide polymorphism; G, genus; RNT, rank-normal transformation; HB, hurdle binary.

## Data Availability

The datasets used and/or analyzed in the current study are available from the corresponding author on reasonable request.

## Supplementary Materials

The following supporting information can be downloaded at: https://www.mdpi.com/article/10.3390/genes14010156/s1, Supplementary File S1: Supplement to the neuroticism score questions; Table S1: Interactions between individual SNPs and gut microbiota for neuroticism with *P* < 5 × 10^–8^; Table S2: Interactions between individual SNPs and gut microbiota for general happiness with *P* < 5 × 10^–8^; Table S3: List of neuroticism-associated GO terms; Table S4: List of general happiness-associated GO terms.
