# Supplementary material for: Evaluating the Genetic Effects of Gut Microbiota on the Development of Neuroticism and General Happiness: A Polygenic Score Analysis and Interaction Study Using UK Biobank Data
Source: Genes (Basel). 2023 Jan 6;14(1):156. doi: 10.3390/genes14010156 (PMC9858947; doi:10.3390/genes14010156)
Supplement: Supplementary file 1 [file genes-14-00156-s001.zip › Supplementary File S1.pdf]

Supplementary File S1: Supplement to the neuroticism score questions.

Neuroticism score questions included:

- Does your mood often go up and down?
- Do you ever feel 'just miserable' for no reason?
- Are you an irritable person?
- Are your feelings easily hurt?
- Do you often feel 'fed-up'?
- Would you call yourself a nervous person?
- Are you a worrier?
- Would you call yourself tense or 'highly strung'?
- Do you worry too long after an embarrassing experience?
- Do you suffer from 'nerves'?
- Do you often feel lonely?
- Are you often troubled by feelings of guilt?
